# Distinct effects of heterogeneity and noise on gamma oscillation in a model of neuronal network with different reversal potential

**DOI:** 10.1038/s41598-021-91389-8

**Published:** 2021-06-21

**Authors:** Tianyi Zheng, Kiyoshi Kotani, Yasuhiko Jimbo

**Affiliations:** 1 Graduate School of Engineering, The University of Tokyo, Tokyo, 113-8656 Japan; 2 Research Center for Advanced Science and Technology, The University of Tokyo, Tokyo, 153-8904 Japan

**Keywords:** Computational neuroscience, Statistical physics, thermodynamics and nonlinear dynamics

## Abstract

Gamma oscillation is crucial in brain functions such as attentional selection, and is inextricably linked to both heterogeneity and noise (or so-called stochastic fluctuation) in neuronal networks. However, under coexistence of these factors, it has not been clarified how the synaptic reversal potential modulates the entraining of gamma oscillation. Here we show distinct effects of heterogeneity and noise in a population of modified theta neurons randomly coupled via GABAergic synapses. By introducing the Fokker-Planck equation and circular cumulants, we derive a set of two-cumulant macroscopic equations. In bifurcation analyses, we find a stabilizing effect of heterogeneity and a nontrivial effect of noise that results in promoting, diminishing, and shifting the oscillatory region, and is largely dependent on the reversal potential of GABAergic synapses. These findings are verified by numerical simulations of a finite-size neuronal network. Our results reveal that slight changes in reversal potential and magnitude of stochastic fluctuations can lead to immediate control of gamma oscillation, which would results in complex spatio-temporal dynamics for attentional selection and recognition.

## Introduction

Synchronization is widely observed in many natural and artificial systems^1,2^. In the field of neuroscience, gamma oscillation (30–200 Hz, including the higher gamma range), binding with gamma-band synchronization, is observed in the cerebral cortex and hippocampus, related to different cognitive functions^3–7^. It is known that a GABAergic (gamma-aminobutyric acid) neuronal population plays important roles in generating gamma oscillation^8–11^ and abnormality of GABAergic neurons alters gamma oscillation in diseases such as epilepsy^12,13^, autism^14^ and schizophrenia^15^. One important feature of gamma oscillation is sparse firings of individual neurons, in which the oscillatory state is close to an asynchronous state and a large proportion of neurons do not fire even at the peak of the gamma cycle^16,17^. It is generally believed that both neuronal heterogeneity and noise play important roles in achieving such moderate synchronization under synaptic interactions^11^. Although heterogeneity and noise are naively considered to destroy the synchronized state, the two factors are not incorporated at the same time because of mathematical difficulties. Thus, most theoretical and numerical studies consider separately either heterogeneity^7,18–21^ or noise^22–25^. Therefore, little is known about how each factor affects gamma oscillation, especially under physiologically plausible changes in synaptic interactions.

In this study, we focused on the interneuron gamma (ING) state that is known as one possible mechanism of gamma oscillations observed in experiment^26^. ING is generated by a population of interneurons coupled via GABAergic synapses, and excitatory neurons do not actively contribute to the generation of gamma oscillation^11,27^. we consider a population of voltage-dependent theta neurons^23^ coupled via GABAergic synapses in which both heterogeneity and noise are incorporated together. Note that although GABA is a primary inhibitory neurotransmitter, but in our study, whether the synapse increase or decrease membrane potential of connected neurons is controlled by reversal potential. The GABAergic synaptic reversal potential, which is known to take values typically from $-50$ to $-90$ mV^28,29^, can change significantly during development^30^, past activities^29^, dynamics of membrane transport proteins^28^, and control synchrony of neuronal oscillations^31^. We adopt a modified theta (MT) model to describe individual neurons, in which appropriate synaptic interactions can be analyzed mathematically^23^. By utilizing reduction theory based on the circular cumulant^32^, we derive a set of low-dimensional equations for macroscopic dynamics. Then we investigate the distinct roles of heterogeneity and noise with different values of reversal potential.

## Model

We start with a neuronal population described by the MT model with heterogeneity and noise. The MT model is a physiologically precise version of the theta model^23^. The phase of the *i*-th MT neuron satisfies the following differential equation:

1 $$\begin{aligned} C\frac{d\theta _{i}}{dt}=-g_{L}\cos \theta _{i}+\frac{2}{V_{T}-V_{R}}(1+\cos \theta _{i})(I_{i}+\sigma \xi _{i}(t))+g^{i}_{syn}\left[ \frac{2V_{syn}-V_{T}-V_{R}}{V_{T}-V_{R}}(1+\cos \theta _{i})-\sin \theta _{i}\right] , \end{aligned}$$

where $C=1(\upmu {\mathrm{F/cm}}^2)$ is the membrane capacitance, $g_L=0.1(\upmu {\mathrm{S/cm}}^2)$ is the leak conductance, $V_{T}=-55({\mathrm{mV}})$ is the firing threshold, $V_{R}=-62({\mathrm{mV}})$ is the resting potential, and $V_{syn}$ is the reversal potential of GABAergic synaptic currents. The entire term in square bracket serves as coupling function. By changing the value of $V_{syn}$, the sign of the term in square bracket will also change. If the whole term in square bracket is positive, then the effect of synapse is to increase the membrane potential of the *i*-th neuron, otherwise decrease the membrane potential. We note that the phase $\theta _{i}$ is transformed from a version of quadratic integrate-and-fire neuron model (QIF) and the membrane potential of *i*-th neuron can be evaluated by $V_{i}=\frac{V_{T}+V_{R}}{2}+\frac{V_{T}-V_{R}}{2}\tan \frac{\theta _{i}}{2}$ (see “Methods” for details). $\xi _{i}(t)$ represents noise with $\langle \xi _{i}(t)\rangle =0$ and $\langle \xi _{i}(t),\xi _{i}(t')\rangle =\delta _{ij}\delta (t-t')$, and $\sigma$ is the magnitude of noise. $I(\upmu {\mathrm{A/cm}}^{2})$ represents the input current. To employ two-cumulant truncation, following previous studies^32–34^, we adopt a Cauchy-Lorentz distribution $r(I)=\frac{1}{\pi }\frac{\Delta }{(I-\eta )^2+\Delta ^2},$ as the distribution of input currents, where $\eta$ and $\Delta$ are the center and width of the distribution, respectively. $\Delta$ is the scale of heterogeneity, with larger $\Delta$ for larger heterogeneity in the neuronal population. The *i*-th neuron fires when $\theta _i$ exceeds $\pi$ and modulates the membrane potential of the connected neuron by GABAergic synapses. $g^{i}_{syn}$ represents the dynamics of GABAergic synaptic conductance. With mean-field approximation of the random and sparse connectivity, the dynamics of conductance obeys the following equation:

2 $$\begin{aligned} \frac{dg^{i}_{syn}}{dt}=-\frac{1}{\tau _{d}}g^{i}_{syn}+{\bar{g}}_{peak}\cdot P_{syn}\cdot N\cdot A(t), \end{aligned}$$

where $\tau _{d}=5(ms)$ is the decay time constant, ${\bar{g}}_{peak}=0.0214~({\mathrm{mS/cm}}^{2})$ is peak conductance, *N* is the number of neurons in the neuronal population. In the numerical simulation of finite neurons, we set $N=3000$, which is considered to be an appropriate size for a typical layer within a single column^35^. $P_{syn}$ is the probability of random synaptic connections between neurons. *A*(*t*) is the firing rate of the neuronal population. Note that Eq. (2) is derived by mean-field approximation of the initial starting point (see “Methods” for details). Equations (1) and (2) constitute the microscopic model for numerical simulation as well as the starting point for deriving the macroscopic model.

For the derivation of a macroscopic model, firstly, we derive the Fokker-Planck equation (FPE) to describe the state of an infinite size neuronal network, and expand the probability density function (PDF) of FPE in a Fourier series. Next, we introduce the circular cumulant referred to in the novel dimension reduction method proposed and obtain the first two cumulants with the smallness assumption^32^. Since the $\sigma$ term in Eq. (1) is multiplicative, which is additive in the reference paper, some modifications are required. (For a step-by-step derivation, see “Methods”.) Then, the two-cumulant macroscopic model is derived as,

3a $$\begin{aligned} {\dot{Z}}= & {} if(Z^{2}+\kappa )+ihZ+if^{*}-c_{3}\sigma ^{2}[(Z+1)^{3}+3\kappa (Z+1)], \end{aligned}$$

3b $$\begin{aligned} {\dot{\kappa }}= & {} 4ifZ\kappa +2ih\kappa -c_{3}\sigma ^{2}[(Z+1)^{4}+12\kappa (Z+1)^{2}+9\kappa ^{2}], \end{aligned}$$

where $f=\frac{1}{2C}[-g_{L}+c_{1}{(\eta +i\Delta )}+c_{2}g_{syn}+ig_{syn}]$, $f^{*}=\frac{1}{2C}[-g_{L}+c_{1}(\eta +i\Delta )+c_{2}g_{syn}-ig_{syn}]$, $h=\frac{1}{C}[c_{1}{(\eta +i\Delta )}+c_{2}g_{syn}]$, $c_{1}=2/(V_{T}-V_{R})$, $c_{2}=(2V_{syn}-V_{T}-V_{R})/(V_{T}-V_{R})$, $c_{3}=c_{1}^2/(4C^{2})$, *i* is an imaginary unit, and $*$ denotes a complex conjugate. *Z* and $\kappa$ are the first and second order cumulants, respectively. Note that Eq. (3a) is in a form of the addition of four terms, that the first three terms are noise-free terms and the last term represents the effect of noise. If we set $\kappa =0$, the first three terms are exactly same as the dimension reduction result of the Ott-Antonsen Ansatz^36^. The firing rate *A*(*t*) of the macroscopic model can also be derived,

4 $$\begin{aligned} A(t)=\frac{g_{L}}{C\pi }\left\{ \frac{1-\vert Z(t)\vert ^{2}}{2\vert 1+Z(t)\vert ^{2}}+Re\left[ \frac{\kappa (t)}{(1+Z(t))^{3}}\right] \right\} . \end{aligned}$$

Equations (2), (3) and (4) constitute the entire macroscopic model with two cumulants *Z* and $\kappa$. To begin with the analysis of this macroscopic model, we first describe the main analysis and validation idea of this paper. Our focus is whether the firing rate *A*(*t*) is stable or not. Although it is hard to analyze the probability density function in the Fokker-Planck equation Eq. (9) itself, we can analyze the equilibrium point and its stability of the two-cumulant macroscopic model derived in our study [Eqs. (2), (3) and (4)]. When the linearized equation around the equilibrium point has only negative eigenvalues in the real part, the firing rate *A*(*t*) is also stable in time. It undergoes a Hopf bifurcation when a pair of eigenvalues cross the imaginary axis due to certain parameter changes. This bifurcation finally results in the oscillation of the firing rate *A*(*t*). Since we consider five parameters in all in the macroscopic model ($\eta$, $V_{syn}$, $P_{syn}$, $\Delta$, $\sigma$), in order to scrutinize the effect of $\Delta$ and $\sigma$, we plot 2-dimension bifurcation diagrams using two of the three variables ($\eta$, $V_{syn}$, $P_{syn}$) and study the changing curves due to the changing of $\Delta$ and $\sigma$. In the validation part, we compare the bifurcation diagrams of the macroscopic model with the results of numerical simulations of finite neurons ($N=3000$) based on the microscopic model [Eqs. (1) and (2)]. All bifurcation diagrams of two-cumulant macroscopic model are plotted with XPPAUT^37^. All numerical simulations of the microscopic model are integrated by the Euler method, with a time step $\Delta t=0.01$ (ms), using MATLAB (2019b, http://www.mathworks.com/products/matlab/).

## Results

In the primary research, we investigated the Hopf bifurcation with respect to the single variable $\eta$ (Supplementary Material Fig. S1). Since $\eta$ determines the center of distribution of input current *I*, increasing $\eta$ could increase the average input current to neurons, thus activate the system. With different parameter settings, the system could achieve one of two states in the long run: stationary state or oscillatory state. We also showed the transition between two states by gradually increasing $\eta$ with time in Supplementary Material Fig. S2, which reflects that the behavior of neuronal network turned from stationary state to oscillatory state in a certain value of the parameter.

### Distinct roles of heterogeneity and noise in ($\eta$, $V_{syn}$) plane

**Figure 1:**
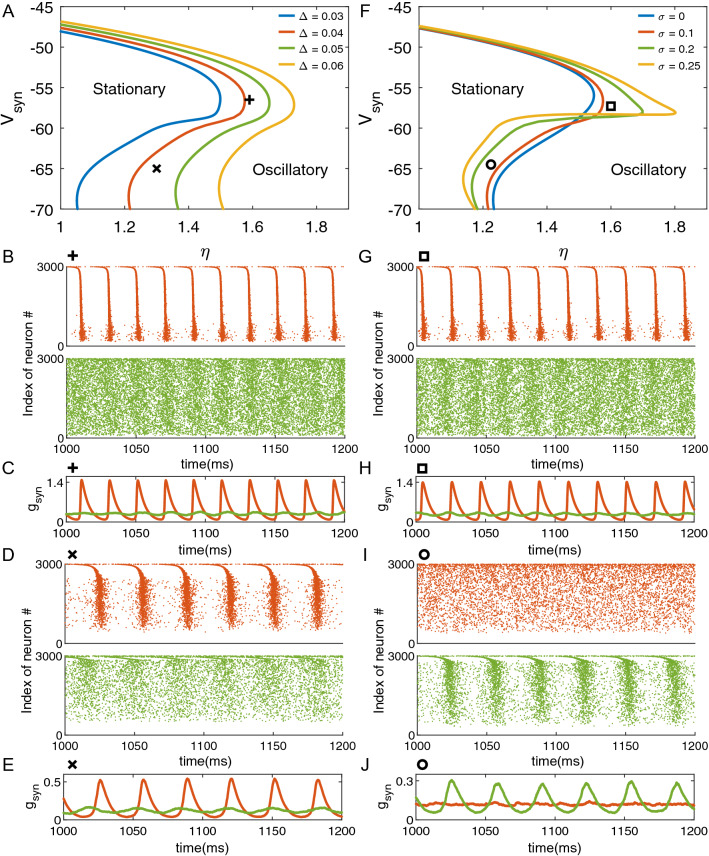
Distinct roles of heterogeneity and noise in ($\eta$, $V_{syn}$) plane (**A**), (**F**) 2-D Bifurcation diagrams of the two-cumulant model [Eqs. (2), (3) and (4)] with different $\Delta$ or $\sigma$. The two regions marked “Stationary” and “Oscillatory” correspond to different dynamic modes: the region of the stationary state and that of the limit-cycle oscillation, respectively. The plus sign, multiplication sign, square and circle denote four different positions on the parameter plane. (**A**) The four curves are all plotted under $P_{syn}=0.1, \sigma =0.1$, but with different values of heterogeneity $\Delta$. (**F**) The four curves are all plotted under $P_{syn}=0.1, \Delta =0.04$, but with different values of heterogeneity $\sigma$. (**B**), (**D**), (**G**), (**I**) Raster plot of four marked positions, obtained from numerical simulation [Eqs. (1) and (2)]. (**C**), (**E**), (**H**), (**J**) Time-courses of $g_{syn}$ at four marked positions, obtained from numerical simulation [Eqs. (1) and (2)]. All bifurcation diagrams of macroscopic model are plotted with XPPAUT^37^. All numerical simulations of the microscopic model are generated by MATLAB (2019b, http://www.mathworks.com/products/matlab/).

First, we analyze different roles of heterogeneity and noise in the ($\eta$, $V_{syn}$) plane, as shown in Fig. 1A and 1F. The plane is divided into two regions by each curve: the region of the stationary state and that of the limit-cycle oscillation corresponding to the state with high gamma oscillation power. The curve itself serves as the boundary of two different states. Figure 1A shows the role of heterogeneity. When we keep the magnitude of noise $\sigma =0.1$ fixed, with increasing heterogeneity $\Delta$, the curve moves rightward, leading to the enlargement of the stationary region. With any specific $V_{syn}$, larger heterogeneity $\Delta$ always tends to enlarge the stationary region of $\eta$. This enlargement effect is weakened with the increase of $V_{syn}$, given that in the rightward movement of the curves in the lower half-plane is more obvious than in the upper half-plane. The range of $\Delta$ and $\sigma$ are set in 0.03–0.06 and 0–0.25 separately. In terms of $\Delta$, the changing of bifurcation diagrams is monotonic, so we only choose several values to show the tendency. In terms of $\sigma$, the range of $\sigma$ is limited by two-cumulant truncation, because if $\sigma$ is too large, cumulant higher than order two cannot be ignored.

Next, we employ numerical simulation of finite neurons by [Eqs. (1) and (2)] to reveal the dynamics of single neurons and neuronal population. Positions marked with a plus sign and a multiplication sign are both on the right side of the red curve ($\Delta =0.04$), which is the oscillatory side, and on the left side of the green curve ($\Delta =0.05$), which is the stationary side. Figure 1B contains raster plots showing the behavior of all 3000 neurons, obtained by numerical simulation, under [$\eta =1.59$, $V_{syn}=-56.5$, plus sign]. In order to eliminate the influence of the initial condition, the raster plots and time-courses are segments of simulation starting from 1000 (ms). In Fig. 1B, the raster plot with red dots corresponds to the behavior under $\Delta =0.04$ at the position marked with a plus sign, and the one with green dots is the behavior under $\Delta =0.05$ at the same position in the parameter plane. Note that the conditions of the raster plot in Fig. 1B match those in the same color in Fig. 1A. Such color matching is also applied to Fig. 1C–E and Fig. 1G–J. The raster plots show that when the parameter setting is on the oscillatory side of the bifurcation curve, neurons tend to fire synchronously. However, when the parameter setting is on the stationary side of the bifurcation curve, neurons fire asynchronously, which seems like random firing on the raster plot. Figure 1C is the time-courses of $g_{syn}$ by numerical simulation of finite neurons, under [$\eta =1.59$, $V_{syn}=-56.5$, plus sign]. Figure 1C, D, it is clearly shown that the time-courses of the same position with different $\Delta$ result in different states, very low amplitude oscillation (green curves) or gamma oscillation (red curves), which show excellent correspondence with the regional division in the bifurcation diagram in Fig. 1A. The raster plot Fig. 1D and the time-course Fig. 1E also agree with the regional division in Fig. 1A. The fluctuation close to stationary state (green curves) is due to the finite size effect because we assume the neuronal network has an infinite size in the derivation of the macroscopic two-cumulant model, while setting 3000 neurons in microscopic numerical simulation. The finite size effect could be regarded as extra noise of the mean field of order $\sim N^{-1/2}$, where *N* is the size of finite ensemble^38^. In the stationary region close to the bifurcation curve, since there are always two conjugate eigenvalues close to imaginary axis, the extra drive by finite size effect yields small-amplitude resonance. In the oscillatory region, extra fluctuation by finite size effect makes the oscillation on each cycle slightly different with each other.

The effect of noise in the ($\eta$, $V_{syn}$) plane is shown in Fig. 1F. We keep heterogeneity $\Delta =0.04$ fixed, with the increase in the magnitude of noise $\sigma$. Unlike the simple moving effect of heterogeneity shown in Fig. 1A, the change in curves due to $\sigma$ is more complicated and largely dependent on $V_{syn}$. For small $V_{syn}$ in the lower half-plane, a larger magnitude of noise $\sigma$ tends to shrink the stationary region of $\eta$, while for large $V_{syn}$, a larger magnitudes of noise $\sigma$ tends to enlarge the stationary region of $\eta$. For even larger $V_{syn}$, an increasing $\sigma$ seems to have no effect on the stationary region. Figure 1G contains raster plots of [$\eta =1.6$, $V_{syn}=-57.3$, square], which clearly shows synchronous or asynchronous activity under different magnitude of noise $\sigma$. Comparing Fig. 1B with 1G, we can observe that two directions to stabilize oscillation (increasing $\Delta$ or $\sigma$) show similar raster plots, at least in the vicinity of bifurcation. Figure 1H is the time-courses of numerical simulation of finite neurons, under [$\eta =1.6$, $V_{syn}=-57.3$, square] . The square is on the right side of the red curve ($\sigma =0.1$) and on the left side of the green curve, while the situation marked by the circle is exactly opposite. Figure 1G,H show that small noise gives rise to the gamma oscillation (red), and large noise gives rise to the stationary state (green). However, in Fig. 1I,J, small noise stabilizes the oscillation (red), while large noise arouses high amplitude oscillation (green). The raster plot and time-courses in Fig. 1I,J also agree with the result of the bifurcation diagram in Fig. 1F.

Besides, we investigated the effect of firing threshold ($V_{T}$) in Supplementary Material Fig. S3. The bifurcation analysis shows that increasing firing threshold monotonically stabilizes the neuronal network. This result is in agreement with intuition since a higher firing threshold means harder to fire for single neurons. We also use numerical simulation of 3000 neurons to show that no matter the reversal potential higher or lower the firing threshold, the above result is correct.

**Figure 2:**
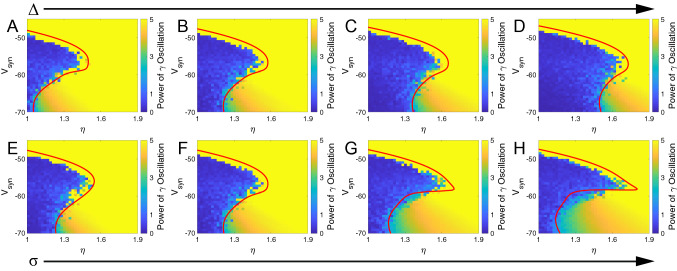
Validation of the bifurcation analyses in ($$\eta$$, $$V_{syn}$$) plane. The red curve in each figure is the boundary of the stationary state region and limit-cycle oscillation state region, obtained from the two-cumulant macroscopic model [Eqs. (2), (3) and (4)]. The heatmap background in each figure represents the power of gamma oscillation $[\uppercase {P}_{\gamma}']$ obtained from the numerical simulation of the microscopic model [Eqs. (1) and (2)]. Note that (**B**) and (**F**) are the same figures with [$$\Delta =0.04$$, $$\sigma =0.1$$] serving as the home position. (**A**)–(**D**) represent the increase in $$\Delta$$. (**E**)–(**H**) represent the increase in $$\sigma$$. All numerical simulations of the microscopic model are generated by MATLAB (2019b, http://www.mathworks.com/products/matlab/).

**Figure 3:**
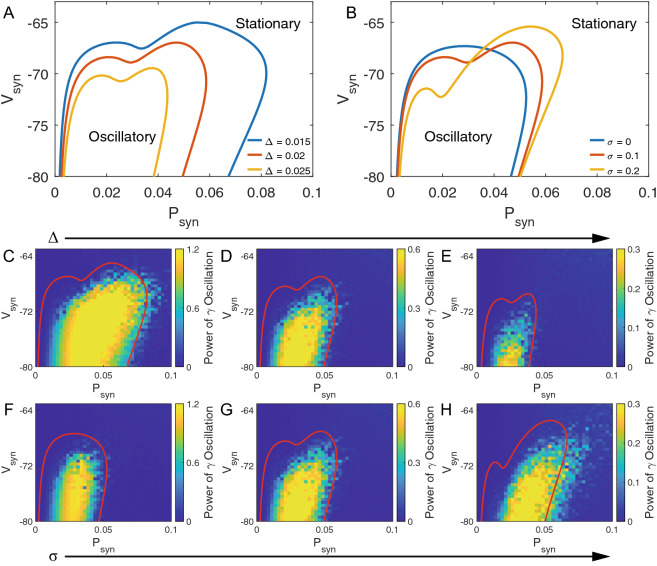
Distinct roles of heterogeneity and noise in ($P_{syn}$, $V_{syn}$) plane (**A**) Role of heterogeneity in ($P_{syn}$, $V_{syn}$) plane. Bifurcation diagram of the two-cumulant model [Eqs. (2), (3) and (4)]. The three curves are all plotted under [$\eta =0.7, \sigma =0.1$], but with different values of heterogeneity $\Delta$: 0.015 for blue, 0.02 for red, 0.025 for yellow. (**B**) The role of noise in the ($P_{syn}$, $V_{syn}$) plane. The three curves are all plotted under [$\eta =0.7, \Delta =0.02$], but with different values of magnitude of noise $\sigma$: 0 for blue, 0.1 for red, 0.2 for yellow. (**C**)–(**E**) Validation of increasing $\Delta$. (**F**)–(**H**) Validation of increasing $\sigma$. All bifurcation diagrams of macroscopic model are plotted with XPPAUT^37^. All numerical simulations of the microscopic model are generated by MATLAB (2019b, http://www.mathworks.com/products/matlab/).

In order to further validate the results of the bifurcation diagram obtained from the two-cumulant macroscopic model [Eqs. (2), (3) and (4)], we compare it with a heatmap showing the power of gamma oscillation at each point on the plane obtained from numerical simulation of finite neurons based on a microscopic model [Eqs. (1) and (2)]. To obtain the total frequency power, we perform a fast Fourier transform to the time-course of $[g_{syn}]$ and integrate the power spectral density over the frequency domain of gamma oscillation (30–200 Hz). To normalize the power difference between stationary points and oscillatory points and avoid negative value, we adjust the integration of power spectral density $\uppercase {P}_{\gamma }$ by introducing a transformation $\uppercase {P}_{\gamma }'=\ln (10^{4}\uppercase {P}_{\gamma }+1)$. The value used to plot heatmaps in Figs. 2 and 3 is the integration of $\uppercase {P}_{\gamma }'$ over the frequency band of gamma oscillation (30–200 Hz). The power spectrum is showed in Supplementary Material Fig. S4. Fig. 2A–D represent the effect of increasing heterogeneity $\Delta$, with values: 0.03, 0.04, 0.05 and 0.06, respectively. Figures 2E–H represent the effect of increasing magnitude of noise $\sigma$ with values: 0, 0.1, 0.2, 0.25, respectively. The parameter settings in Figs. 2A–D are same as in Figs. 1A, 2E–H are same as in Fig. 1F. Although there are some randomly scattered dots near the boundary of the two regions due to the effect of noise in the numerical simulation, these heatmaps and curves achieve an excellent agreement, indicating the correctness of the bifurcation diagrams shown in Fig. 1A,F. Besides, we found that the center frequency of oscillation monotonically increases with $V_{syn}$ in a wide frequency range, which includes both lower gamma and higher gamma range. (Supplementary Material Fig. S4).

### Distinct roles of heterogeneity and noise in ($P_{syn}$, $V_{syn}$) plane

We next investigate the role of heterogeneity and noise in the ($P_{syn}$, $V_{syn}$) plane, and consider the rate of random synaptic connection $P_{syn}$. Figure 3A represents the effect of heterogeneity $\Delta$. When we keep the magnitude of noise $\sigma =0.1$ fixed, with the increase of heterogeneity $\Delta$, the regions inside the curves shrink, which enlarges the stationary state region, similar to Fig. 1A. With any given $V_{syn}$, the oscillatory region of $P_{syn}$ monotonically shrinks. Figure 3B shows the role of noise in ($P_{syn}$, $V_{syn}$) plane. When we keep the heterogeneity $\Delta =0.02$ fixed, shown as Fig. 3B, with increasing magnitude of noise $\sigma$, the curves twist and regions rotate. By further analyzing the nontrivial rotation, it is shown that the oscillatory regions of $P_{syn}$ are dependent on $V_{syn}$. When increasing $V_{syn}$, the oscillatory regions of $P_{syn}$ move rightward, while keeping the area of oscillatory region.

The validation of bifurcation analyses in the ($P_{syn}$, $V_{syn}$) plane is shown in Figs. 3C–E and F–H, which are plotted by the same method as Fig. 2. Figure 3C–E represent the effect of increasing heterogeneity $\Delta$ with values: 0.015, 0.02, 0.025. Figure 3F–H represent the effect of increasing magnitude of noise $\sigma$ with values: 0, 0.01, 0.02. Note that Fig. 3C,G are the same figures under [$\Delta =0.02$, $\sigma =0.1$], serving as the home position. Considering heterogeneity $\Delta$ alone, the agreement of heatmaps and curves in Fig. 3A,C–E is excellent, showing the gradually shrinking oscillatory region with increasing $\Delta$. In terms of magnitude of noise $\sigma$, shown as Fig. 3B and F–H, one observes a clear rotation phenomenon of the oscillatory region in the heatmaps just as predicted by the bifurcation curves. This means that in some regions, gamma oscillation emerges with increasing noise, while in some other regions it stabilizes. Although there is some considerable difference between bifurcation diagrams and heatmaps due to finite size effect of numerical simulation, especially in Fig. 3H, the qualitative rotation effect can be clearly observed.

Besides ($\eta$, $V_{syn}$) plane and ($P_{syn}$, $V_{syn}$) plane, we also investigated ($\eta$, $P_{syn}$) in Supplementary Material Fig. S5, by bifurcation analysis of the two-cumulant macroscopic model [Eqs. (2), (3) and (4)] and numerical simulation of microscopic model [Eqs. (1) and (2)]. The result showed an agreement between macroscopic and microscopic models. Moreover, we confirmed that, similar to other bifurcation analysis (Figs. 1 and 3), the effect of $V_{syn}$ is complex, which cannot be simply classified as stabilizing or facilitating oscillations.

## Discussion

In this work, we formulated a set of macroscopic low-dimensional differential equations from an ensemble of spiking neuron models that enable us to analyze the entraining of gamma oscillation. The strength of our model is that it possesses voltage-dependent dynamics and analytically bridges micro-macro dynamics for gamma oscillations considering both noise and heterogeneity. Moreover, unlike numerical simulation of a large number of neurons by microscopic model, since our two-cumulant model is analytical, it explicitly shows the effect of each parameter and don’t suffer from numerical issues such as computational cost. Though relatively complicated in the form, to some extent, our two-cumulant macroscopic model can mechanistically explain the reason behind phenomenon. In Supplementary Material Section 6, we analyzed how $\Delta$ suppresses the oscillation by changing the eigenvalue of the Jacobian matrix of the system and found that it is similar to the effect of heterogeneity in Kuramoto model^1,2^. Instead, the rotation in Fig. 3B is really nontrivial and is found using our macroscopic equations in this study.

In research into synchronization of general coupled oscillators, both heterogeneity of the oscillator ensemble and induced noise have shown significant effects on synchronization. According to some previous research, heterogeneity suppresses synchronization^39^, while noise promotes the onset of synchronization in some cases^40,41^. In this work on neuronal populations, similar to previous research, heterogeneity diminishes macroscopic oscillation (Figs. 1A and 3A). The role of noise is largely dependent on synaptic reversal potential $V_{syn}$, which is closely related to the coupling function in general coupled oscillators. With a different setting of $V_{syn}$, noise promotes, suppresses or changes the region of synchronization (Figs. 1F and 3B). Therefore, our model demonstrates that even small changes in the coupling function can alter the effect of noise in collective dynamics.

As the MT model incorporates voltage dependent membrane dynamics and synaptic interactions in a physiologically plausible manner, we obtain macroscopic Eq. (3) with somewhat harder computation than previous research. Ratas et al.^33^ analyzed macroscopic dynamics of quadratic integrate-and-fire neurons. When considering both heterogeneity and noise together, they simply put forward the argument that effects of heterogeneity and noise are topologically similar in a bifurcation diagram. However, in our work, we found that the effect of heterogeneity and noise on gamma oscillation is different. Heterogeneity of a neuronal network stabilizes the oscillation. The reversal potential only monotonically affects the extent of this stabilization, but no qualitative effect. Nevertheless, the effect of noise is qualitatively dependent on reversal potential. Comparing with the work from Ratas et al., there are several differences that need to be mentioned. The first is we adopt a more detailed single neuron model with reversal potential $V_{syn}$, which has been reported to contribute to the synchronous discharges for epilepsy in an *in vitro* experiment^13^. $V_{syn}$ determines whether the synapse increase or decrease membrane potential in our model.The second is they only consider excitatory neurons, while we varied the value of reversal potential within the experimentally observed range for GABAergic neurons. It basically decreases, but can increase in some cases, the membrane potential of the connected neuron. Thirdly, their single neuronal model is close to the firing threshold, which is not our case.

Gamma oscillation in a neuronal population plays important roles in brain functions such as attentional selection^3,42^, signal discrimination^43,44^ and learning^45^. Therefore, controlling gamma oscillation is functionally required. Especially in the case of attentional selection, it is required for the higher visual cortex to shift the gamma synchronized target immediately to the attended area of the lower V1 population^42^. The reversal potential $V_{syn}$, that is physiologically altered by intracellular calcium concentration through calcium-dependent ${\mathrm{Cl}}^{-}$ transporters^28,29^, largely altered the dynamics of neuron.In our model, since the membrane potential is typically changing between the resting potential $V_{R}$ and the firing threshold $V_{T}$, if $V_{syn}<V_{R}$, the synapse would always decrease the membrane potential. On the other hand, if $V_{syn}>V_{T}$, the synapse would always increase the membrane potential. This qualitative effect of $V_{syn}$ on synapse also shapes the bifurcation curve. Our results further reveal that $V_{syn}$ leads to immediate control of the gamma oscillation and synchronization. We also note that stochastic fluctuation in the membrane potential is increased by acetylcholine^46,47^, thus $\sigma$ is also considered to vary more rapidly than other properties of the neuronal network. From these points, our results bridge gamma oscillation and ion channel, and imply that the dynamics of gamma oscillation can be well controlled by slight changes (in mV or sub-mV order) of $V_{syn}$ and $\sigma$, which have a potential influence on attentional selection and other cognitive functions. Because gamma oscillation exhibits complex spatio-temporal dynamics^3,4,6^ and both reduced and excess oscillation are found in diseases such as autism^14^ and schizophrenia^15^, our analyses shed light on another influence of the reversal potential and stochastic fluctuations on brain functions through gamma oscillation.

## Methods

### Transformation from quadratic integrate-and-fire model to modified theta model

The quadratic integrate-and-fire (QIF) model is a typical model for class I neurons near the firing threshold^48^. The dynamics of the membrane potential of the *i*-th neuron satisfy the following equation:

5 $$\begin{aligned} C\frac{dV_{i}}{dt}=g_{L}\frac{(V_{i}-V_{R})(V_{i}-V_{T})}{V_{T}-V_{R}}-g^{i}_{syn}(V_{i}-V_{syn})+I_{i}+\sigma \xi _{i}(t), \end{aligned}$$

where $V_{T}=-55$ is the firing threshold, and $V_{R}=-62$(mV) is the resting potential. The sign of $V_{i}-V_{syn}$ determines whether the synapse increases or decreases the membrane potential: when negative, it increases the membrane potential, otherwise decreasing the membrane potential. The original form of $g_{syn}^{i}(t)$ in Eqs. (1) and (5) obeys the following first-order equation:

6 $$\begin{aligned} \frac{dg^{i}_{syn}}{dt}=-\frac{1}{\tau _{d}}g^{i}_{syn}+{\bar{g}}_{peak}\sum _{k=1}\delta (t-t^{(k)}), \end{aligned}$$

where ${\bar{g}}_{peak}=0.0214({\mathrm{mS/cm}}^{2})$ is obtained by dividing peak conductance of GABA on interneurons (6.2 nS)^52^ by membrane surface area of neuron ($2.9\times 10^{-4}\, {\mathrm{cm}}^{2}$)^53^. $\delta (\cdot )$ is the Dirac delta function and $t^{(k)}$ is the time of firing of pre-synaptic neurons connected to *i*-th neuron. With mean-field approximation, the sum of the Dirac delta function can be replaced, which transforms to Eq. (2).

In order to avoid the membrane potential value $V_{i}(t)$ jumping from $+\infty$ to $-\infty$ when firing, a phase variable $\theta _{i}(t)$ can be introduced in Eq. (5),

7 $$\begin{aligned} V_{i}=\frac{V_{T}+V_{R}}{2}+\frac{V_{T}-V_{R}}{2}\tan \frac{\theta _{i}}{2}, \end{aligned}$$

where $-\pi <\theta \le \pi$. This variable transformation turns the QIF model into a modified theta model^23^. Compared with the infinite value of $V_{i}(t)$, the phase value $\theta _{i}(t)$ simply crosses the value of $\theta _{i}(t)=\pi$ at these firing moments. Eq. (5) can be transformed into Eq. (1).

### Derivation of two-cumulant model

Here, we elaborate on details of the derivation from the microscopic model [Eqs. (1) and (2)] of the two-cumulant macroscopic model [Eqs. (2), (3) and (4]. The dynamics of phase oscillator ${\dot{\theta }}$ in Eq. (1) can be separated into a deterministic part *u* and a stochastic part *w*:

8a $$\begin{aligned} d\theta&=udt+w\circ dW, \end{aligned}$$

8b $$\begin{aligned} u(\theta ,I,t)&=\frac{1}{C} \{ -g_{L}\cos \theta +c_1I(1+\cos \theta )+g_{syn}\left[ c_{2}(1+\cos \theta )-\sin \theta \right] \}, \end{aligned}$$

8c $$\begin{aligned} w(\theta ,I,t)&=\frac{1}{C}c_1\sigma (1+\cos \theta ), \end{aligned}$$

where $\circ dW$ is the (Stratonovich) white noise differential^49^. In the thermodynamic limit of an infinite oscillator ensemble ($N\rightarrow \infty$), its state can be described by $\rho (\theta ,I,t)$ which satisfies the Fokker-Planck equation (FPE). Note that the multiplicative form of the stochastic parameter (with the Stratonovich interpretation) in our model Eq. (8c) is different from the additive noise term in the reference paper^32^. The corresponding FPE in our case is obtained as^50^

9 $$\begin{aligned} \frac{\partial \rho }{\partial t}=-\frac{\partial }{\partial \theta }\left\{ \left[ u+\frac{w}{2}\frac{\partial w}{\partial \theta }\right] \rho \right\} +\frac{1}{2}\frac{\partial ^2}{\partial \theta ^2}(w^2\rho ). \end{aligned}$$

It is helpful to separate the AC and DC parts of *u* at this stage as

10a $$\begin{aligned} u(\theta ,I,t)&=f(I,t)e^{i\theta }+h(I,t)+f(I,t)^{*}e^{-i\theta }, \end{aligned}$$

10b $$\begin{aligned} f(I,t)&=\frac{1}{2C}[-g_{L}+c_{1}I+c_{2}g_{syn}+ig_{syn}], \end{aligned}$$

10c $$\begin{aligned} h(I,t)&=\frac{1}{C}[c_{1}I+c_{2}g_{syn}]. \end{aligned}$$

Expanding $\rho (\theta ,I,t)$ in a Fourier series in $\theta$, we have

11 $$\begin{aligned} \rho (\theta ,I,t)=\frac{r(I)}{2\pi }\left\{ \alpha _{0}+\left[ \sum _{j=1}^{\infty }\alpha _{j}(I,t)e^{-ij\theta }+c.c.\right] \right\} , \end{aligned}$$

where $\alpha _{0}=1$ and *c*.*c*. stands for complex conjugate. Applying Eq. (11) to Eq. (9) and comparing the exponent of *e* on both sides of the equation, we obtained an infinite series of the complex amplitude of a Fourier mode,

12 $$\begin{aligned} \dot{\alpha _{j}}=ijf\alpha _{j+1}+ijh\alpha _{j}+ijf^{*}\alpha _{j-1}-\frac{1}{2}c_3\sigma ^2\left[ 6j^2\alpha _{j}+(4j^2+2j)\alpha _{j+1}+(4j^2-2j)\alpha _{j-1}+(j^2+j)\alpha _{j+2}+(j^2-j)\alpha _{j-2}\right] , \end{aligned}$$

where $c_{3}=c_{1}^2/(4C^{2})$, $j\ge 1$ and $\alpha _{-1}=0$. We note that the amplitude $\alpha _{j}=\int _{-\pi }^{\pi }\rho (\theta ,I,t)e^{ij\theta }d\theta$ is the Kuramoto–Daido order parameters^51^ at a given *I*. Next, we integrate $\alpha _{j}(I,t)$ over the distribution *r*(*I*) as

13 $$\begin{aligned} Z_{j}(t)=\int _{-\infty }^{\infty }r(I)\alpha _{j}(I,t)dI. \end{aligned}$$

Then, on the assumption that $\alpha _{j}(I,t)$ is analytic in the upper half-plane of the complex variable *I*^36^, the integral can be computed by the residue on the upper half-plane as

14 $$\begin{aligned} Z_{j}(t)=\alpha _{j}(\eta +i\Delta ,t). \end{aligned}$$

As a result, we substitute $\dot{Z_{j}}$ for $\dot{\alpha _{j}}$ in Eq. (12) and obtain an infinite series of ordinary differential equations for the order parameter $Z_{j}(t)$,

15 $$\begin{aligned} \dot{Z_{j}}=ijfZ_{j+1}+ijhZ_{j}+ijf^{*}Z_{j-1}-\frac{1}{2}c_3\sigma ^2\left[ 6j^2Z_{j}+(4j^2+2j)Z_{j+1}+(4j^2-2j)Z_{j-1}+(j^2+j)Z_{j+2}+(j^2-j)Z_{j-2}\right] , \end{aligned}$$

where $Z_{j}$ series start from $j=1$, and set $Z_{0}=1$, $Z_{-1}=0$.

In order to obtain a set of low-dimensional reduced equations of the system Eq. (15), we follow a novel method^32^ based on circular cumulants. Order parameters $Z_{j}=\langle e^{ij\theta }\rangle$ can be regarded as the *j*th moment of the random variable $e^{i\theta }$, which are determined by a moment-generating function

16 $$\begin{aligned} F(k,t)=\langle \exp (ke^{i\theta })\rangle \equiv \sum _{j=0}^{\infty }Z_{j}(t)\frac{k^{j}}{j!}. \end{aligned}$$

Thus, the related order parameters and its time derivatives can be written as,

17 $$\begin{aligned} Z_{j}=\left. \frac{\partial ^{j}}{\partial k^{j}}F(k,t)\right| _{k=0},\frac{\partial F}{\partial t}=\sum _{j=0}^{\infty }\dot{Z_{j}}(t)\frac{k^{j}}{j!}. \end{aligned}$$

Substituting *F*(*k*, *t*) for $Z_{j}(t)$ in Eq. (15) using Eq. (17), and comparing the exponent of *k* on both sides of the equation, the partial differential equation for *F*(*k*, *t*) follows

18 $$\begin{aligned} \frac{\partial F}{\partial t}&= ifk\frac{\partial ^2F}{\partial k^2}+ihk\frac{\partial F}{\partial k}+if^{*}kF \nonumber \\&\quad -\frac{1}{2}c_3\sigma ^2\left[ 6k\frac{\partial }{\partial k}\left( k\frac{\partial F}{\partial k}\right) +4k\frac{\partial }{\partial k}\left( k\frac{\partial ^2 F}{\partial k^2}\right) +2k\frac{\partial ^2F}{\partial k^2}+4k\frac{\partial }{\partial k}(kF)\right. \nonumber \\ {}&\quad \left. -2kF+k\frac{\partial }{\partial k}\left( k\frac{\partial ^3 F}{\partial k^3}\right) +k\frac{\partial ^3 F}{\partial k^3}+k^2F \right] . \end{aligned}$$

The circular cumulants introduced in^32^ are determined by a cumulant-generating function defined as

19 $$\begin{aligned} \psi (k,t)=k\frac{\partial }{\partial k}\ln F=\frac{k}{F}\frac{\partial F}{\partial k}\equiv \sum _{j=1}^{\infty }\chi _{j}(t)k^{j}. \end{aligned}$$

From Eqs. (16) and (19), one can derive the relationship between order parameters $Z_{j}(t)$ and circular cumulants $\chi _{j}(t)$. For the first two cumulants,

20 $$\begin{aligned} \chi _{1}=Z_{1},\quad \chi _{2}=Z_{2}-Z_{1}^{2}. \end{aligned}$$

Applying $\partial _{t}$ to $\psi$ in Eq. (19), we obtain the relationship of $\frac{\partial F}{\partial t}$ and $\frac{\partial \psi }{\partial t}$ as following

21 $$\begin{aligned} \frac{\partial \psi }{\partial t} = -\frac{1}{F}\psi \frac{\partial F}{\partial t}+\frac{k}{F}\frac{\partial }{\partial k}\left( \frac{\partial F}{\partial t}\right) . \end{aligned}$$

Exerting Eq. (18) into Eq. (21), the partial differential equation for $\psi (k,t)$ can be derived as

22 $$\begin{aligned} \frac{\partial \psi }{\partial t}&=ifk\frac{\partial }{\partial k}(kA)+ihk\frac{\partial \psi }{\partial k}+if^{*}k-c_3\sigma ^2\left[ 3k\frac{\partial }{\partial k}\left( k\frac{\partial \psi }{\partial k}+\psi ^{2}\right) +k\frac{\partial }{\partial k}\left( kA\right) +(-2k\psi ^{2}+4k\psi +k+k^2) \right. \nonumber \\&\quad \left. +A(2k^3+2k-2k\psi )+B(6k^2+k-2k^2\psi -k\psi )+ C\left( 2k^3+2k^2-\frac{1}{2}k^2\psi \right) +\frac{1}{2}k^3D\right] , \end{aligned}$$

where *A*, *B*, *C*, *D* contain the second, third, fourth, fifth partial derivatives of *F*(*k*, *t*) to *t*:

23a $$\begin{aligned} A&=\frac{1}{F}\frac{\partial ^2F}{\partial k^2}=\frac{\partial }{\partial k}\left( \frac{\psi }{k}\right) +\frac{\psi ^2}{k^2}, \end{aligned}$$

23b $$\begin{aligned} B&=\frac{1}{F}\frac{\partial ^3F}{\partial k^3}=\frac{\partial }{\partial k}\left( A\right) +\frac{\psi }{k}A, \end{aligned}$$

23c $$\begin{aligned} C&=\frac{1}{F}\frac{\partial ^4F}{\partial k^4}=\frac{\partial }{\partial k}\left( B\right) +\frac{\psi }{k}B, \end{aligned}$$

23d $$\begin{aligned} D&=\frac{1}{F}\frac{\partial ^5F}{\partial k^5}=\frac{\partial }{\partial k}\left( C\right) +\frac{\psi }{k}C. \end{aligned}$$

The complete form of $\psi$ is shown in Eq. (26). On the assumption that the smallness of the third cumulant is $O(\sigma ^{4})$, which vanishes in an approximation^32^, we only take the first two cumulants into consideration. By applying $\psi (k,t)=\chi _{1}(t)k+\chi _{2}(t)k^{2}$ to Eq. (22), we finally obtain the first two cumulants in the macroscopic model Eq. (3), where $\chi _{1}=Z$ denotes the first cumulant and $\chi _{2}=\kappa$ denotes the second cumulant. In order to achieve an explicit form of firing rate *A*(*t*) in Eq. (2), which is determined as

24 $$\begin{aligned} A(t)=\left. \int _{-\infty }^{+\infty }\left( u+\frac{w}{2}\frac{\partial w}{\partial \theta }\right) \rho \right| _{\theta =\pi } dI, \end{aligned}$$

one requires an explicit form of $\rho (\theta ,I,t)$ first, coming from Eq. (11). With the approximation of only two cumulants, the moment-generating function *F*(*k*, *t*) in Eq. (16) can be simplified as $F=\exp [kZ+\kappa (k^{2}/2)]$. Following the assumption^32^ that $F\approx [1+\kappa (k^{2}/2)]\exp [kZ]$ under smallness of $\kappa$, the moments $Z_{j}$ can be derived by performing $\partial _{t}$ to *F* as $Z_{j}=Z^{j}+[j(j-1)/2]\kappa Z^{j-2}$. Apply $Z_{j}$ to Eq. (11), and the summation of Fourier series is

25 $$\begin{aligned} \rho (\theta ,I,t)=\frac{r(I)}{2\pi }\left\{ \frac{1-|Z|^2}{|e^{i\theta }-Z|^2}+2Re\left[ \frac{\kappa e^{i\theta }}{(e^{i\theta }-Z)^3}\right] \right\} . \end{aligned}$$

The firing rate *A*(*t*) can be written in an explicit form by applying Eq. (25) to Eq. (24), shown as Eq. (4).

### Complete form of cumulant-generating function $\psi$

The complete form of $\psi$ of Eq. (22) is

26 $$\begin{aligned}&\frac{\partial \psi }{\partial t}=ifk\frac{\partial }{\partial k}\left[ k\frac{\partial }{\partial k}\left( \frac{\psi }{k}\right) +\frac{\psi ^2}{k}\right] +ihk\frac{\partial \psi }{\partial k}+if^{*}k-c_{3}\sigma ^{2}\left\{ 3k\frac{\partial }{\partial k}\left( k\frac{\partial \psi }{\partial k}+\psi ^{2}\right) +(-2k\psi ^{2}+4k\psi +k+k^2) \right. \nonumber \\&\quad \left. +k\frac{\partial }{\partial k}\left[ k\frac{\partial }{\partial k}\left( \frac{\psi }{k}\right) +\frac{\psi ^2}{k}\right] +\left[ \frac{\partial }{\partial k}\left( \frac{\psi }{k}\right) +\frac{\psi ^2}{k^2}\right] (2k^3+2k-2k\psi )\right. \nonumber \\&\quad \left. +\left\{ \frac{\partial }{\partial k}\left[ \frac{\partial }{\partial k}\left( \frac{\psi }{k}\right) +\frac{\psi ^2}{k^2}\right] +\frac{\psi }{k}\left[ \frac{\partial }{\partial k}\left( \frac{\psi }{k}\right) +\frac{\psi ^2}{k^2}\right] \right\} (6k^2+k-2k^2\psi -k\psi ) \right. \nonumber \\&\quad +\left\{ \frac{\partial }{\partial k}\left\{ \frac{\partial }{\partial k}\left[ \frac{\partial }{\partial k}\left( \frac{\psi }{k}\right) +\frac{\psi ^2}{k^2}\right] +\frac{\psi }{k}\left[ \frac{\partial }{\partial k}\left( \frac{\psi }{k}\right) +\frac{\psi ^2}{k^2}\right] \right\} +\frac{\psi }{k}\left\{ \frac{\partial }{\partial k}\left[ \frac{\partial }{\partial k}\left( \frac{\psi }{k}\right) +\frac{\psi ^2}{k^2}\right] \right. \right. \nonumber \\&\quad \left. \left. +\frac{\psi }{k}\left[ \frac{\partial }{\partial k}\left( \frac{\psi }{k}\right) +\frac{\psi ^2}{k^2}\right] \right\} \right\} \cdot \left( 2k^3+2k^2-\frac{1}{2}k^2\psi \right) \nonumber \\&\quad +\frac{1}{2}k^{3}\frac{\partial }{\partial k}\left\{ \frac{\partial }{\partial k}\left\{ \frac{\partial }{\partial k}\left[ \frac{\partial }{\partial k}\left( \frac{\psi }{k}\right) +\frac{\psi ^2}{k^2}\right] +\frac{\psi }{k}\left[ \frac{\partial }{\partial k}\left( \frac{\psi }{k}\right) +\frac{\psi ^2}{k^2}\right] \right\} +\frac{\psi }{k}\left\{ \frac{\partial }{\partial k}\left[ \frac{\partial }{\partial k}\left( \frac{\psi }{k}\right) +\frac{\psi ^2}{k^2}\right] \right. \right. \nonumber \\&\left. \left. \quad +\frac{\psi }{k}\left[ \frac{\partial }{\partial k}\left( \frac{\psi }{k}\right) +\frac{\psi ^2}{k^2}\right] \right\} \right\} +\frac{1}{2}k^{3}\frac{\psi }{k}\left\{ \frac{\partial }{\partial k}\left\{ \frac{\partial }{\partial k}\left[ \frac{\partial }{\partial k}\left( \frac{\psi }{k}\right) +\frac{\psi ^2}{k^2}\right] +\frac{\psi }{k}\left[ \frac{\partial }{\partial k}\left( \frac{\psi }{k}\right) +\frac{\psi ^2}{k^2}\right] \right\} \right. \nonumber \\&\left. \left. \quad +\frac{\psi }{k}\left\{ \frac{\partial }{\partial k}\left[ \frac{\partial }{\partial k}\left( \frac{\psi }{k}\right) +\frac{\psi ^2}{k^2}\right] +\frac{\psi }{k}\left[ \frac{\partial }{\partial k}\left( \frac{\psi }{k}\right) +\frac{\psi ^2}{k^2}\right] \right\} \right\} \right\} . \end{aligned}$$

## Supplementary Information

Supplementary Information.
