# Supplementary material for: Distinct effects of heterogeneity and noise on gamma oscillation in a model of neuronal network with different reversal potential
Source: Sci Rep. 2021 Jun 21;11:12960. doi: 10.1038/s41598-021-91389-8 (PMC8217259; doi:10.1038/s41598-021-91389-8)
Supplement: Supplementary file 1 — Supplementary Information. [file 41598_2021_91389_MOESM1_ESM.pdf]

Distinct Effects of Heterogeneity and Noise on Gamma Oscillation in a Model of Neuronal Network with Different Reversal Potential: Supplementary Material

1. SINGLE VARIABLE BIFURCATION DIAGRAM

In primary analysis, the bifurcation diagram with only respect to η is analyzed. Since η determines the center of distribution of input current I, increasing η could increase the average input current to neurons thus activate the system. Fig. S1A shows the bifurcation diagram of single variable η (center of the distribution of input current). The system undergoes Hopf bifurcation when $\eta = 1.245$ marked as red star, turning from stationary state into oscillatory state. Figs. S1B-S1F are time-courses under five different setting of η marked on Fig. S1A: [$\eta = -0.146$, blue], [$\eta = 0$, green], [$\eta = 0.143$, cyan], [$\eta = 1.141$, yellow], [$\eta = 1.333$, purple]. Although differences exist in unstable state, e.g. first 500ms in Fig S1E and first 1000ms in S1F, the time-courses obtained from two-cumulant model (gray) achieve excellent agreement with numerical simulation of finite neurons (Colored) in long time stable state.

The Blue, green, cyan dots describe the system performance in the vicinity of $\eta = 0$, where the distribution of input current is symmetrical. Under such a small average input current, the firing rate of neurons is low thus cannot reach a synchronous state. Although increasing η changes the ratio of negative to positive values of input current, it only results in a gradual increase of the average g_{syn} in stationary state. Yellow and purple dots describe the system performance close to Hopf bifurcation: before the bifurcation, the system eventually goes to stationary state, while after bifurcation the system shows stable oscillation in the long run. Considering trivial performance close to $\eta = 0$ and enlarge the changing of bifurcation point, for the remainder of this paper, we focus on the case $\eta > 1$.

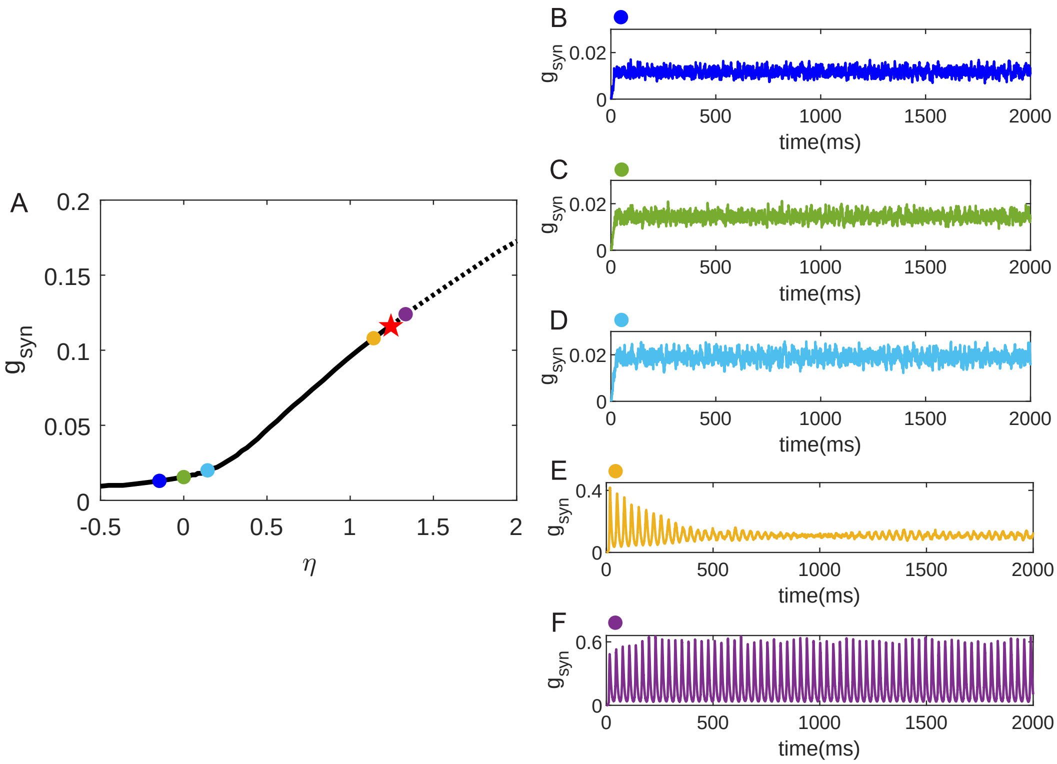

Fig. S1. Single variable bifurcation analysis(η). (**A**) Bifurcation diagram of single variable η. Other fixed variables are $V_{syn} = -65$, $P_{syn} = 0.1$, $\Delta = 0.04$, $\sigma = 0.1$. The red star represents the occurrence of Hopf bifurcation. The solid line represents stationary state, while the dot line represents oscillatory state. Five colored dots represent that the system under five different settings of η. (**B**)-(**F**) Time-courses of five corresponding marked place in (**A**). The color line in each figure is obtained by numerical simulation [Eqs. (1) and (2)]. The gray line in each figure is obtained by the macroscopic two-cumulant model [Eqs. (2), (3) and (4)] with XPPAUT [1]. The numerical simulation of the microscopic model is generated by MATLAB (2019b, http://www.mathworks.com/products/matlab/).

.

2. TRANSITION FROM STATIONARY STATE TO OSCILLATORY STATE

In order to show the transition from stationary state to oscillatory state in numerical simulation of finite size neuronal network, we increase η (center of the distribution of input current) with time, as $\eta(t) = \eta_0 + \mu t$, where $\eta_0 = 1$ is starting value of the simulation, μ is the step of η increasing with time. Since we set the end value as $\eta = 1.4$ and simulation time as $t = 10^6$(ms), the step should be $\mu = (1.4 - 1)/10^6 = 4 \times 10^{-7}(/\text{ms})$.

Fig. S2A shows the parameter setting of this experiment. This red bifurcation curve is the same as red curves in Figs. 1A and 1F. We fix $V_{syn} = -65$ as the blue arrow positioned, and gradually increase η with time. Figs. S2B and S2C shows that the behavior of neuronal network has qualitative change near $\eta = 1.2$ which is close to the intersection of red curve and blue curve in Fig. S2A. Before this bifurcation point, the neuronal network showing stationary activity with very low fluctuation. After bifurcation, the neuronal network showing clear oscillatory activity and the amplitude of oscillation increase with increasing of η.

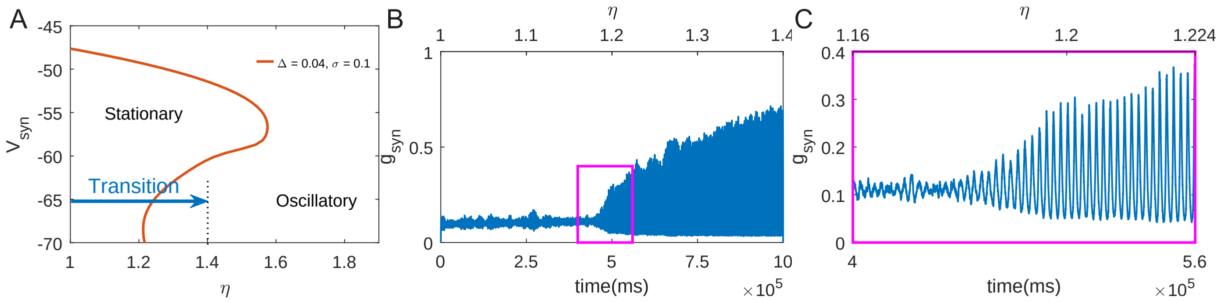

Fig. S2. Transition from stationary state to oscillatory state. (**A**) Bifurcation diagram of the two-cumulant model [Eqs. (2), (3) and (4)] with $P_{syn} = 0.1$. The blue arrow labeled as "Transition" is the simulation route. (**B**) Time-course of g_{syn} obtained from numerical simulation [Eqs. (1) and (2)] showing the transition. (**C**) the zoom-in view of the magenta box in (**B**). All bifurcation diagrams of macroscopic model are plotted with XPPAUT [1]. The numerical simulation of the microscopic model is generated by MATLAB (2019b, http://www.mathworks.com/products/matlab/).

.

3. BIFURCATION ANALYSIS IN (η, V_{syn}) PLANE UNDER DIFFERENT FIRING THRESHOLD V_T

We investigated the effect of firing threshold (V_T) in (η, V_{syn}) plane. Fig. S3A is the bifurcation diagram under different V_T, where the red curve is the same as red curves in Fig. 1A and 1F. Higher V_T monotonically enlarge stationary region and stabilize the oscillation. We also pick two positions between red curve and green curve: [$\eta = 1.6$, $V_{syn} = -53$, star sign] and [$\eta = 1.6$, $V_{syn} = -56$, dot sign]. The reversal potential for star position is higher than the firing threshold, thus modeled as synapses increasing membrane potential. Conversely, the reversal potential for dot position is lower than the firing threshold, which is modeled as synapses decreasing membrane potential. Both positions exhibit similar behavior which agrees with the bifurcation diagram, showing that no matter the synapses increasing or decreasing membrane potential, the stabilization effect exists.

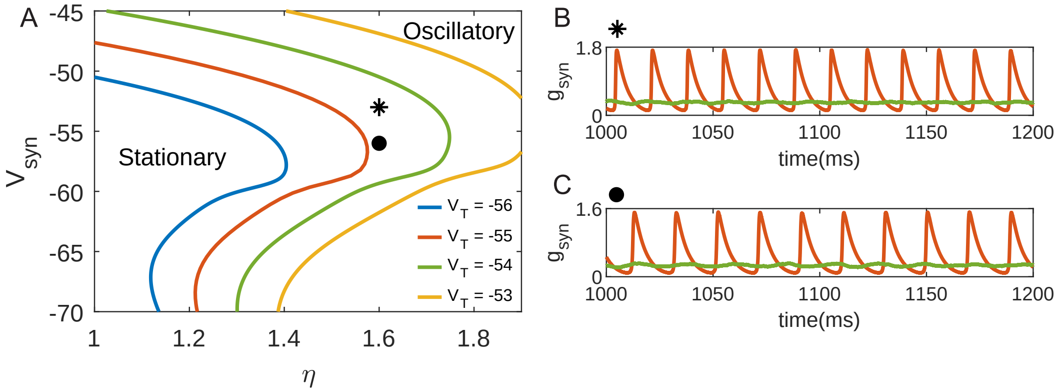

Fig. S3. Bifurcation analysis in (η, V_{syn}) plane under different firing threshold V_T. (**A**) Bifurcation diagram of the two-cumulant model [Eqs. (2), (3) and (4)] with different V_T. The red curve serves as home position [$\Delta = 0.04$, $\sigma = 0.1$, $V_{syn} = -70$]. (**B**)(**C**) are time-courses of corresponding marked position in (**A**), obtained by numerical simulation [Eqs. (1) and (2)]. The bifurcation diagram of macroscopic model is plotted with XPPAUT [1]. All numerical simulations of the microscopic model are generated by MATLAB (2019b, http://www.mathworks.com/products/matlab/).

.

4. POWER SPECTRUM AND CENTER FREQUENCY OF OSCILLATIONS

We investigated the power spectrum and center frequency of oscillation in Fig. S2. We pick a position in the oscillatory region and marked as an upward-pointing triangle [$\eta = 1.7154$, $V_{syn} = -55.2564$] as Fig. S4A showed. The corresponding time-course of triangle position is Fig. S4B. By performing a fast Fourier transform to the time-course, we obtain a power spectrum as Fig. S4C. There is a clear center frequency in the gamma band (30–200 Hz, including the higher gamma band). We also investigate how the center frequency will change with changing of reversal potential (V_{syn}) in Fig. S4D. The result shows that the center frequency monotonically increases with the increasing of reversal potential.

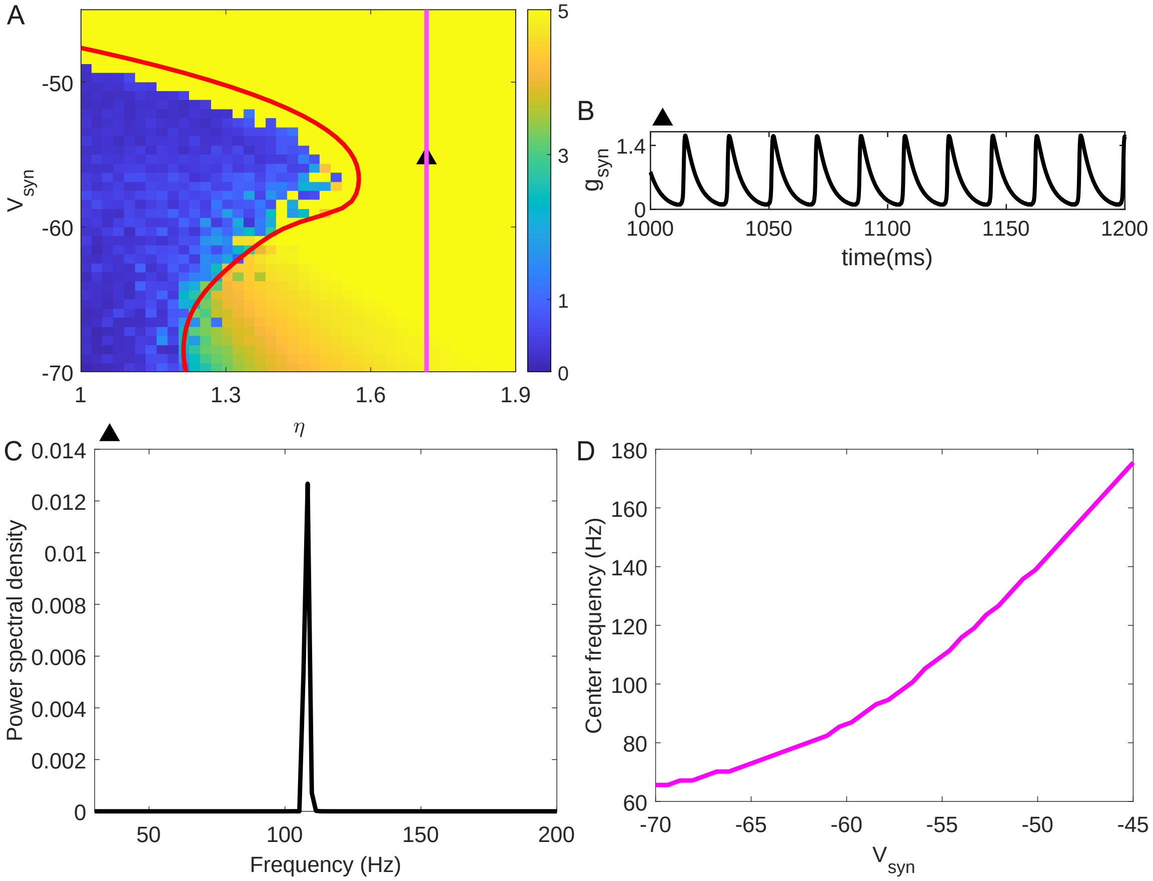

Fig. S4. Power spectrum and center frequency of oscillations. (**A**) The heatmap and red bifurcation curve are the same as Fig. 2B under [$\Delta = 0.04$,$\sigma = 0.1$]. We show the time-course and power spectrum of the position marked as upward-pointing triangle in (**B**)(**C**), by numerical simulation of microscopic model [Eqs. (1) and (2)]. The magenta line indicates the parameter setting increasing V_{syn} while fixed η. (**D**) The center frequency of oscillation increases while increasing V_{syn} and fixed $\eta = 1.7154$. All numerical simulations of the microscopic model are generated by MATLAB (2019b, http://www.mathworks.com/products/matlab/).

5. BIFURCATION ANALYSIS IN (η, P_{syn}) PLANE UNDER DIFFERENT REVERSAL POTENTIAL V_{SYN}

We investigated the effect of reversal potential in (η, P_{syn}) plane. In Fig. S5A, we find that when increasing V_{syn} the bifurcation shows complex changes which cannot be simply classified as stabilizing or facilitating the oscillations. We also picked a position on bifurcation diagram to perform numerical simulation of finite neurons: [$\eta = 0.85$, $P_{syn} = 0.02$, diamond sign]. The time-course in Fig. S5B shows an agreement with the bifurcation diagram.

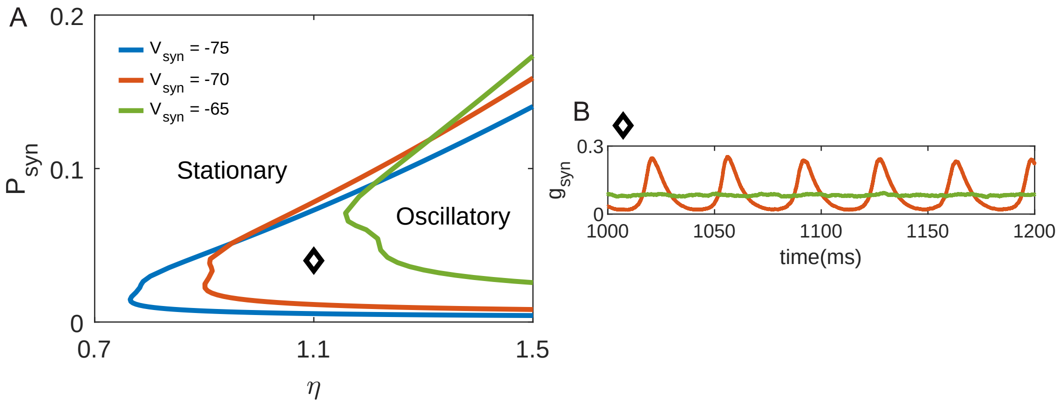

Fig. S5. Bifurcation analysis in (η, P_{syn}) plane. (**A**) Bifurcation diagram of the two-cumulant model [Eqs. (2), (3) and (4)] with different V_{syn}. (**B**) is the time-course of corresponding diamond marked position in (**A**), obtained by numerical simulation [Eqs. (1) and (2)]. The bifurcation diagram of macroscopic model is plotted with XPPAUT [1]. The numerical simulation of the microscopic model is generated by MATLAB (2019b, http://www.mathworks.com/products/matlab/).

.

6. MECHANISM OF HOW INCREASING Δ STABILIZE THE OSCILLATION

In this section, we will describe how the change of Δ gives rise to different states (stationary state and limit-cycle oscillation state). Main idea is to see how eigenvalues of Jacobian matrix changed with Δ. Since Z and κ in the two-cumulant model [Eqs. (2), (3) and (4)] are complex variable, we separate their real part and imaginary part as $Z = Z_1 + iZ_2$ and $\kappa = \kappa_1 + i\kappa_2$. Corresponding ordinary differential equations can be derived by separate real part and imaginary part of Eqs. (2), (3) and (4). The whole system of two-cumulant model consists of a set of five state variables $\mathcal{Q} = \{Z_1, Z_2, \kappa_1, \kappa_2, g_{syn}\}$. The Jacobian matrix J of this system can be obtained by (parameters other than Δ are fixed values)

$$J(\mathcal{Q}, \Delta) = \begin{bmatrix} \frac{\partial \dot{Z}_1}{\partial Z_1} & \frac{\partial \dot{Z}_1}{\partial Z_2} & \frac{\partial \dot{Z}_1}{\partial \kappa_1} & \frac{\partial \dot{Z}_1}{\partial \kappa_2} & \frac{\partial \dot{Z}_1}{\partial g_{syn}} \\ \frac{\partial \dot{Z}_2}{\partial Z_1} & \frac{\partial \dot{Z}_2}{\partial Z_2} & \frac{\partial \dot{Z}_2}{\partial \kappa_1} & \frac{\partial \dot{Z}_2}{\partial \kappa_2} & \frac{\partial \dot{Z}_2}{\partial g_{syn}} \\ \frac{\partial \dot{\kappa}_1}{\partial Z_1} & \frac{\partial \dot{\kappa}_1}{\partial Z_2} & \frac{\partial \dot{\kappa}_1}{\partial \kappa_1} & \frac{\partial \dot{\kappa}_1}{\partial \kappa_2} & \frac{\partial \dot{\kappa}_1}{\partial g_{syn}} \\ \frac{\partial \dot{\kappa}_2}{\partial Z_1} & \frac{\partial \dot{\kappa}_2}{\partial Z_2} & \frac{\partial \dot{\kappa}_2}{\partial \kappa_1} & \frac{\partial \dot{\kappa}_2}{\partial \kappa_2} & \frac{\partial \dot{\kappa}_2}{\partial g_{syn}} \\ \frac{\partial \dot{g}_{syn}}{\partial Z_1} & \frac{\partial \dot{g}_{syn}}{\partial Z_2} & \frac{\partial \dot{g}_{syn}}{\partial \kappa_1} & \frac{\partial \dot{g}_{syn}}{\partial \kappa_2} & \frac{\partial \dot{g}_{syn}}{\partial g_{syn}} \end{bmatrix}.$$

Suppose the system undergoes Hopf bifurcation at $\mathcal{Q}_0$ and Δ_0, the Jacobian matrix obeys $J(\mathcal{Q}_0, \Delta_0)V_0 = V_0 D_0$, where D_0 is the diagonal matrix of corresponding eigenvalues and columns of matrix V_0 are corresponding eigenvectors. Since there must be two conjugate eigenvalues which real part is zero when Hopf bifurcation occurs, let $(D_0)_{1,1}$ and $(D_0)_{2,2}$ represents the eigenvalue on imaginary axis, obeying $Re((D_0)_{1,1}) = Re((D_0)_{2,2}) = 0$. Now consider a slight change in Δ as $\Delta = \Delta_0 + \epsilon$, according to matrix perturbation theory [2] the updated matrix of eigenvalues D_Δ can be approximately obtained by

$$D_\Delta = V_0^{-1} J(\mathcal{Q}_0, \Delta_0) V_0 + V_0^{-1} J(\mathcal{Q}_0, \epsilon) V_0 + O(\epsilon^2) \tag{S6.1}$$

It leads to $\frac{\partial Re((D_\Delta)_{i,i})}{\partial \Delta} = \frac{\partial Re((V_0^{-1} J(\mathcal{Q}_0,\epsilon) V_0)_{i,i})}{\partial \epsilon}$. Thus, the effect of Δ on Hopf bifurcation can be quantitatively measured.

From the analysis by XPPAUT [1], we know the system undergoes Hopf bifurcation under the following parameter setting: $\eta = 1.217$, $V_{syn} = -70$, $P_{syn} = 0.1$, $\Delta_0 = 0.04$, $\sigma = 0.1$ and the values of the set $\mathcal{Q}_0$ are shown as Table. S1.

Table S1. Values of five state variables at Hopf bifurcation.

Z_1	Z_2	κ_1	κ_2	g_{syn}
0.2072	0.4372	0.003992	-0.003451	0.07888

We find that among five eigenvalues of $J(\mathcal{Q}_0, 0.04)$, $\lambda_1(\mathcal{Q}_0, 0.04) = 0 + 0.1678i$ is on the positive side of imaginary axis. Let $\Delta = \Delta_0 + \epsilon$, and employ Eq. (S6.1)

$$Re(\lambda_1(\mathcal{Q}_0, \Delta)) \simeq Re((V_0^{-1} J(\mathcal{Q}_0, \epsilon) V_0)_{1,1})$$

Thus evaluate the partial derivative

$$\frac{\partial Re(\lambda_1(\mathcal{Q}_0, \Delta))}{\partial \Delta} = \frac{\partial Re((V_0^{-1} J(\mathcal{Q}_0, \epsilon) V_0)_{1,1})}{\partial \epsilon} = -0.3621 < 0$$

The increase of Δ will drive $\lambda_1(\mathcal{Q}_0, \Delta)$ into the left half-plane of complex coordinate, which is equivalent to Δ stabilizing the oscillation.

Next, we find Δ appears in both function h and f in the two-cumulant model (Eq. (3)), where h is independent of phase (θ) and f is a function of phase. Therefore, here h acts similar character as the natural frequency in Kuramoto model [3] where each oscillator have its own intrinsic natural frequency. In the case of the Kuramoto model, only the oscillators close to central frequency are synchronized [4], so larger Δ pushes oscillators away

from central frequency and suppresses the synchronization. To confirm that increasing Δ in our model stabilizes the macroscopic gamma oscillations in a similar manner to the Kuramoto model, we evaluate the effect of increasing Δ to disperse the natural firing frequency by only consider h term in the Jacobian matrix. In this case,

$$J^{(h)}(\mathcal{Q}_0, \epsilon) = \begin{bmatrix} -\frac{c_1}{C}\epsilon & 0 & 0 & 0 & 0 \\ 0 & -\frac{c_1}{C}\epsilon & 0 & 0 & 0 \\ 0 & 0 & -\frac{2c_1}{C}\epsilon & 0 & 0 \\ 0 & 0 & 0 & -\frac{2c_1}{C}\epsilon & 0 \\ 0 & 0 & 0 & 0 & 0 \end{bmatrix},$$

then evaluating the corresponding eigenvalue only consider h,

$$\frac{\partial Re(\lambda_1^{(h)}(\mathcal{Q}_0, \Delta))}{\partial \Delta} = \frac{\partial Re((V_0^{-1} J^{(h)}(\mathcal{Q}_0, \epsilon) V_0)_{1,1})}{\partial \epsilon} = -0.2313 < 0,$$

the phase-independent component h of the system has a stabilizing effect on oscillation when increasing Δ.

REFERENCES

1. Ermentrout, G. B. *Simulating, Analyzing, and Animating Dynamical Systems: A Guide to XPPAUT for Researchers and Students*. (Society for Industrial and Applied Mathematics, 2002).
2. Stewart, G., Stewart, J., Sun, J. *Matrix Perturbation Theory*. (Elsevier Science, 1990).
3. Kuramoto, Y. (1975). Self-entrainment of a population of coupled non-linear oscillators *International symposium on mathematical problems in theoretical physics*. (pp. 420-422). Springer, Berlin, Heidelberg.
4. Pikovsky, A., Kurths, J., Rosenblum, M., Kurths, J. *Synchronization: A Universal Concept in Nonlinear Sciences*. (Cambridge University Press, 2003).
